# *Ecytonucleospora hepatopenaei* causes lipid droplet depletion and imbalanced lipid metabolism in *Penaeus vannamei*

**DOI:** 10.1038/s41598-025-21037-y

**Published:** 2025-10-24

**Authors:** Satika Yuanlae, Dararat Thaiue, Sukanya Saedan, Kamonluk Kittiwongpukdee, Rapeepun Vanichviriyakit, Niti Chuchird, Ornchuma Itsathitphaisarn

**Affiliations:** 1 Department of Biochemistry, Faculty of Science, Mahidol University, Rama VI Rd., Bangkok, 10400 Thailand; 2 Center for Excellence in Shrimp Molecular Biology and Biotechnology (Centex Shrimp), Faculty of Science, Mahidol University, Rama VI Rd., Bangkok, 10400 Thailand; 3 Aquatic Animal Health Research Team (AQHT), Integrative Aquaculture Biotechnology Research Group, National Center for Genetic Engineering and Biotechnology (BIOTEC), National Science and Technology Development Agency (NSTDA), Yothi office, Rama VI Rd., Bangkok, 10400 Thailand; 4 Department of Fishery Biology, Faculty of Fisheries, Kasetsart University, 50 Ngamwongwan Rd., Chatuchak, Bangkok, 10900 Thailand; 5 Department of Anatomy, Faculty of Science, Mahidol University, Rama VI Rd., Bangkok, 10400 Thailand

**Keywords:** Molecular biology, Biochemistry

## Abstract

*Ecytonucleospora hepatopenaei* (EHP) infection in *Penaeus vannamei* causes significant economic losses in shrimp aquaculture due to growth retardation. Unable to generate ATP, EHP obligately relies on host energy production to survive. We hypothesize that EHP may potentially disrupt its host’s lipid metabolism as lipid is the most energy-dense resource. To this end, the effects of EHP infection on lipid metabolism were investigated by evaluating activities and expressions of digestive enzymes, accumulation of lipid droplets, and expression of genes involved in lipid breakdown and synthesis. Results revealed that EHP infection progressively diminished digestive enzyme activities, leading to reduced nutrient availability. A persistent reduction of lipid droplet accumulation was detected from the onset of EHP infection. The marked decrease in the early stages was attributable to increased lipid droplet breakdown outpacing lipid synthesis. In the later stage, a partial recovery of lipid synthesis gene expression was observed; however, this was insufficient to counteract increased lipid breakdown, as genes in this pathway were upregulated. Ultimately, this imbalance resulted in a marked depletion of lipid droplets. These findings highlight how EHP infection chronically impairs digestion, drains lipid reservoir of its host and, eventually, retards growth. In terms of a potential practical solution, this study implies that dietary lipid supplementation might alleviate the metabolic disruption of EHP and, thereby, lessen the extent of growth retardation in infected population.

**Supplementary Information:**

The online version contains supplementary material available at 10.1038/s41598-025-21037-y.

## Introduction

Whiteleg shrimp (*Penaeus vannamei*) is one of the most economically significant species in global aquaculture. However, its farming faces significant challenges from various viruses and parasites. Among the most concerning is a microsporidian parasite named *Ecytonucleospora hepatopenaei* (EHP), which causes hepatopancreatic microsporidiosis (HPM). Outbreaks of EHP are prevalent in leading shrimp-farming countries including Thailand, China, Vietnam, India, Malaysia, Indonesia, and Venezuela^1–5^. Notably, while EHP infection does not significantly impact mortality rates, it results in chronic effects that impair shrimp growth and productivity, leading to economic loss^6,7^. Given the economic importance of *P. vannamei* and the threat posed by EHP, a deeper understanding of its infection mechanism and effects on shrimp physiology is crucial. EHP is an obligate intracellular parasite that produces infectious resistant spores and is primarily transmitted via oral route by shrimp ingesting the EHP spores from contaminated feces or infected shrimp tissue^8^. Once ingested, EHP targets specific organs within the shrimp’s digestive system. The primary target organ of EHP is the hepatopancreas (HP), a vital organ in the shrimp digestive system. It comprises three mature cell types: B-cells with large vacuoles for enzyme secretion, digested material transport, and nutrient storage^9^, R-cells (resorptive/reservoir cells) with multiple vacuoles for nutrient absorption and the storage of lipids and glycogen, and F-cells (fibrillar cells) with zymogen granules as precursors of digestive enzymes^10^. The HP is also involved in immunity and metabolism^11^. Although the primary infection site is the HP, the EHP spores are also found in the stomach^12^, which plays an important role in the mechanical and enzymatic breakdown of food, suggesting a broader impact on shrimp digestive health. Additionally, digestive enzyme activity and expression have been shown to be influenced by various physiological conditions including molting and developmental stage of shrimp, body weight, and starvation^13–18^. Previous studies have shown that EHP affects shrimp immunity, metabolism, and growth-related pathways at multiple levels. These effects include transcriptomic, proteomic, and metabolomic changes in the HP and intestine^19–25^. Furthermore, EHP infection alters the composition of hepatopancreatic and intestinal microbiota^26,27^. In addition to these broad effects, EHP infection reduces digestive enzyme activity, including lipase and α-amylase, in the HP^28^. Despite these findings, the specific effects of EHP on digestive enzyme activity in other parts of the digestive tract remain poorly understood. Central to the digestive and metabolic health of shrimp is lipid metabolism, which involves both anabolism (lipid synthesis) and catabolism (lipid breakdown) to maintain cellular energy balance. Under heavy EHP infection, proteins involved in multiple aspects of lipid metabolism, including fatty acid degradation and elongation, synthesis of unsaturated fatty acids, and metabolism of α-linolenic acid, sphingolipids, and glycerolipids, are downregulated^29^. These changes in lipid metabolism are reflected in the altered expression and activity of several key enzymes and regulatory factors. Fatty acid-binding proteins (*FABP*), which facilitate intracellular trafficking of fatty acid, are upregulated during infection^22^. Acetyl-CoA carboxylase 1 (*ACC1*), a rate-limiting enzyme in fatty acid synthesis, is found to be downregulated at the protein level after infection^29^. Fatty acid synthase (*FA*S), another important enzyme in fatty acid synthesis, has shown conflicting responses, with studies reporting both upregulation and downregulation of *FAS* expression^22,30^. Carnitine palmitoyltransferase 1 (*CPT1*), which catalyzes the transport of long-chain fatty acids into mitochondria for beta-oxidation, decreased after EHP infection^29^. In this context, lipid droplets, intracellular organelles that store neutral lipids such as glycerides and sterols, play a crucial role in cellular energy management and host-pathogen interaction^31^. Various intracellular pathogens directly or indirectly manipulate their host’s lipid droplet accumulation and metabolism to meet their own metabolic needs^32^. As an intracellular parasite, EHP may similarly affect lipid droplet dynamics in shrimp. Understanding how EHP affects lipid droplet accumulation and breakdown is particularly pertinent. Lipid droplet breakdown involves a series of enzymes such as pancreatic triacylglycerol lipase (*PTGL*), hormone-sensitive lipase (*HSL*), and monoglyceride lipase (*MGL*), which sequentially hydrolyze stored lipids into free fatty acids and glycerol. Given this intricate relationship between pathogens and host lipid metabolism, a focused examination of how EHP affects lipid droplet dynamics in shrimp is crucial. Despite these findings, it remains unclear how EHP infection influences lipid droplet accumulation and their associated metabolic pathways over time in the shrimp’s digestive organs. We hypothesized that EHP infection leads to a progressive reduction in lipid droplet accumulation within the HP and dysregulates the associated metabolic enzymes. To address this knowledge gap and test our hypothesis, we conducted a laboratory cohabitation assay and examined shrimp at 7-, 14-, and 21-days post-infection. We compared digestive enzyme activity and mRNA expression across multiple digestive tissues in infected versus naïve shrimp. In addition, we measured lipid droplet accumulation, assessed the expression of lipid droplet breakdown-related genes (*PTGL*,* HSL*,* MGL*), and evaluated enzymes involved in lipid transport (fatty acid transport protein (*FATP*) and *FABP*), lipid synthesis *(ACC1 and FAS*) and lipid breakdown (*CPT1*). Our results revealed a reduction in lipid droplet accumulation, altered lipid metabolism, and decreased enzyme activities in EHP-infected shrimp. These observations provide valuable insights into the physiological changes occurring during EHP infection. These findings suggest that the growth retardation observed in EHP-infected shrimp could be attributed to reduced lipid droplet accumulation and an imbalance between lipid synthesis and breakdown in the HP. By addressing the question, we aim to provide new insights into the metabolic disruptions caused by EHP, which could inform more effective management strategies for EHP outbreaks in shrimp aquaculture.

## Results

### EHP infection was observed after cohabitation assay with gradual progression

**Fig. 1:**
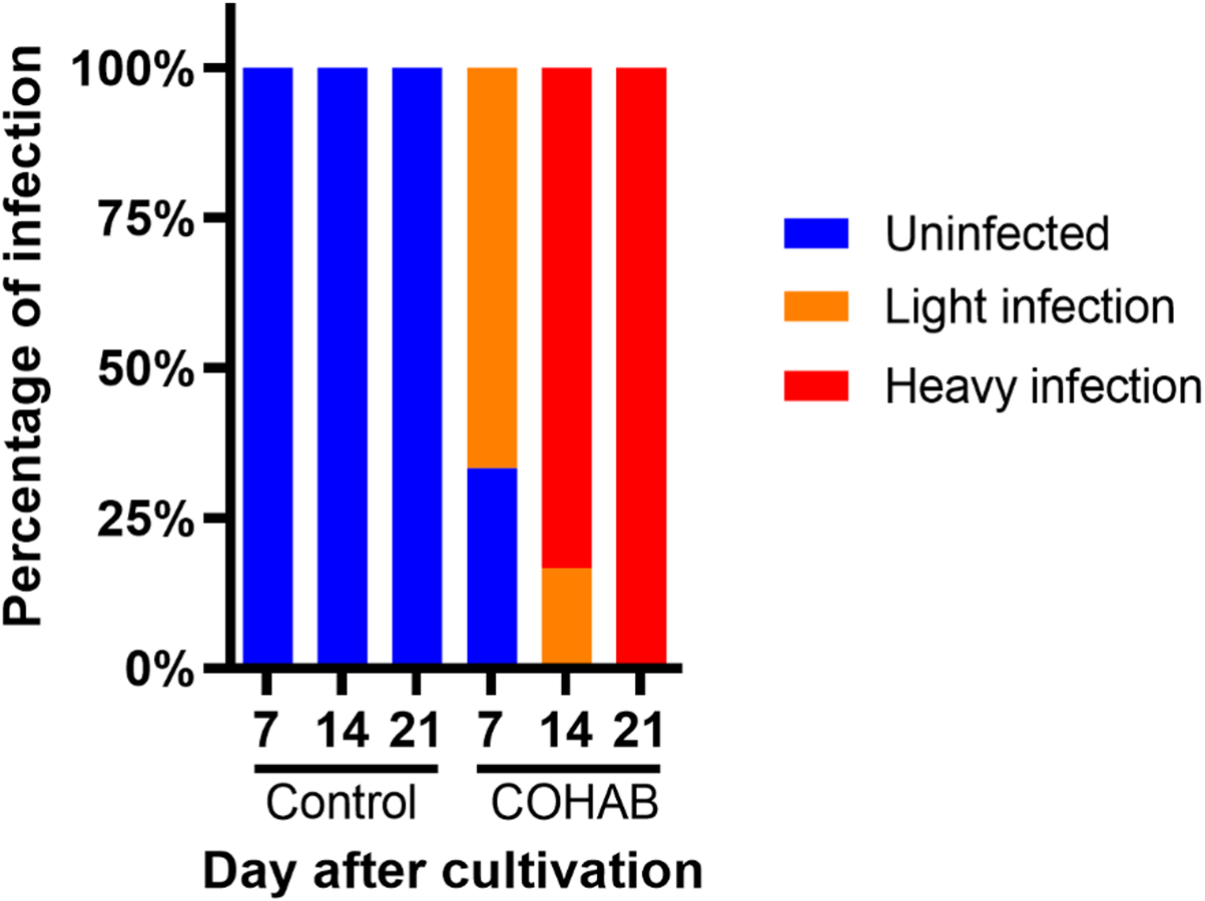
EHP infection occurred in cohabitation assay with gradual progression over 21 days. Shrimp from control and COHAB tanks were tested for EHP infection at 7, 14, and 21 days with nested PCR detecting spore wall protein (SWP). The graph shows the percentage of shrimp in each infection category: uninfected (blue), light infection (orange), and heavy infection (red). EHP infection was classified as heavy if positive PCR products were obtained in both steps, and light if positive only in the second step; uninfected samples were PCR-negative in both steps.

To observe progression of EHP infection in the set-up cohabitation assay, shrimp were randomly collected and examined for EHP infection after 7, 14 and 21 days. This sampling interval was chosen based on our prior experiments (unpublished data), which indicated that shrimp were heavily infected with EHP by 21 days. Therefore, by sampling every 7 days, we aimed to capture the gradual progression of the infection. The results show that there was no EHP infection at any time point in the control tank (Fig. 1). In contrast, shrimp collected from the COHAB tank showed EHP infection at every time point, with gradual progression as expected. These results confirm that EHP can transmit horizontally among shrimp, and the cohabitation assay proceeded as expected.

### EHP infection reduced digestive enzyme activity

Given the known association between EHP infection and stunted growth^28,33,34^, we hypothesized that EHP infection impairs digestive enzyme activity, which then leads to diminishing nutrient absorption and utilization. To test this hypothesis, we examined trypsin, amylase, and lipase activities in the hepatopancreas (HP) and stomach of shrimp taken from control and COHAB tanks over 7, 14, and 21 days. The detection of EHP infection levels in the samples is presented in Supplementary Figures S1−2.

**Fig. 2:**
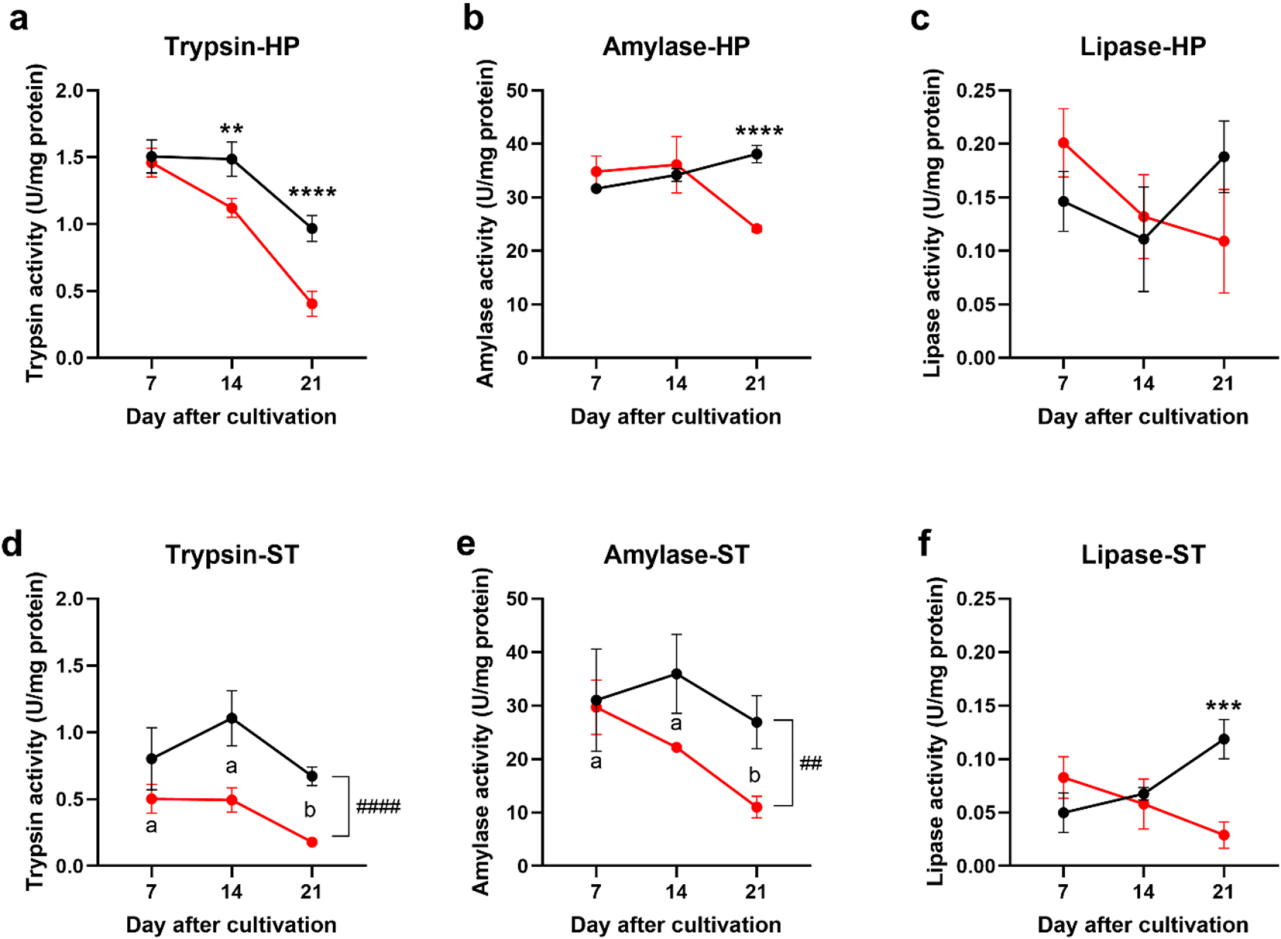
Digestive enzyme activity decreased after EHP infection. Comparison of digestive enzyme activities (trypsin, amylase, and lipase) in the stomach (ST) and hepatopancreas (HP) of shrimp from control (uninfected, black) and COHAB (EHP-infected, red) tanks after 7, 14, and 21 days of cohabitation. Data are presented as mean ± SD (*n* = 3 per group). Two-way ANOVA was used to test effects of time and infection status. For trypsin and amylase in the ST, no significant interaction was detected; significant main effects of time are indicated by different letters (Tukey’s test, comparisons among days averaged across both groups), while hashtags denote main effects of infection status (averaged across time: ## = *p* < 0.01, #### = *p* < 0.0001). For all other cases, significant interactions were detected; asterisks indicate differences between groups at each time point (Šídák’s test: ** = *p* < 0.01, *** = *p* < 0.001, **** = *p* < 0.0001). Enzyme activity is expressed as U/mg protein.

Analysis of enzyme activities revealed disparate patterns across tissues and over time (Fig. 2). Overall, it was found that trypsin, amylase, and lipase activities were higher in the hepatopancreas than the stomach, consistent with previous reports that the digestive enzymes synthesize and accumulate in the HP^9,35^.

Two-way ANOVA revealed significant interactions between time and infection status for all three enzymes in the HP and for lipase in the stomach, indicating that the effect of EHP infection on digestive enzyme activity was time dependent. In these cases, simple effect analyses were performed to examine differences between control and COHAB groups at each time point. No differences were detected between groups on day 7, but from day 14 onwards, infected shrimp exhibited marked reductions: HP trypsin declined by 24.5% at day 14 and 58.3% at day 21 (Fig. 2a); HP amylase was slightly elevated at day 14 but decreased by 36.6% at day 21 (Fig. 2b); and stomach lipase dropped sharply by 75.6% at day 21 (Fig. 2f). HP lipase activity was unaffected by infection across time (Fig. 2c). In addition, analysis of the simple effect of time within each group is shown in Supplementary Figure S3.

For trypsin and amylase in the stomach, where no significant interaction was detected, infection and time effects were evaluated independently. Infection significantly reduced both enzymes overall (averaged across all time points) (Fig. 2d and e). Time also had a significant effect, with Tukey’s post-hoc test showing reductions from day 7 to 21 and day 14 to 21 (averaged across both groups).

Together, these results suggest that EHP infection impacts digestive enzyme activity, particularly with a reduction in later stages of infection (day 14 and 21).

### Digestive enzyme mRNA expression decreased as EHP infection progressed

Digestive enzymes are known to be synthesized in the HP^35^. Therefore, we hypothesized that EHP infection affects the mRNA expression levels of these enzymes in the HP. To explore this, the HP of shrimp collected from the COHAB tank was examined and compared to the control group. The EHP infection levels in the samples are shown in Supplementary Figures S4-5.

**Fig. 3:**
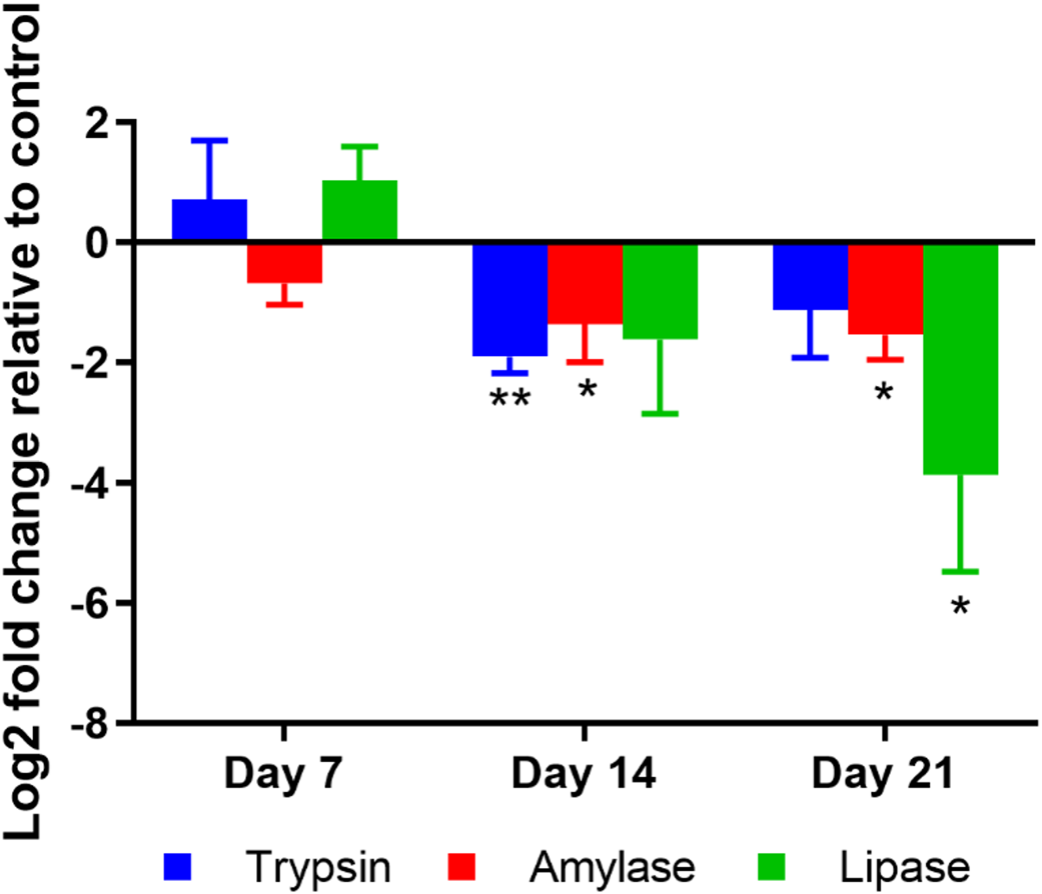
EHP infection decreased mRNA expression of digestive enzymes in hepatopancreas at later stages of infection. The plot illustrates log2-fold change of Trypsin, Amylase and Lipase mRNA expression in hepatopancreas of EHP-infected (COHAB) shrimp after 7, 14, and 21 days of cohabitation, relative to control shrimp at the same time points. Data are presented as mean ± SD (*n* = 3 per group). The asterisks indicate the statistical difference (* = *p* < 0.05, ** = *p* < 0.01) when compared to the control group at the same time point, tested by t-test.

As shown in Fig. 3, there was no significant difference in mRNA expression during the early stages of cultivation (day 7). However, after 14 days of cohabitation, the mRNA expression levels of trypsin and amylase were significantly reduced by 73.0% and 58.5%, respectively. By day 21, amylase and lipase mRNA expression showed marked decreases of 64.7% and 89.8%, respectively, in infected shrimp compared to the control. The findings suggest that EHP infection disrupts the synthesis of these digestive enzymes, particularly as infection progresses.

### Lipid droplets dramatically decreased after EHP infection

Lipid droplet accumulation has been found to be affected by pathogen infection^32^. In this study, we observed a significant reduction in lipase mRNA expression in the HP of shrimp after 21 days of cohabitation. Since lipase plays a crucial role in the hydrolysis of lipids for absorption and storage, this decrease may impair lipid digestion and subsequently hinder lipid storage in the HP. To test the hypothesis, shrimp from the control and COHAB tanks were collected and subjected to Oil Red O staining, allowing for the visualization of lipid droplets in the HP under a light microscope.

**Fig. 4:**
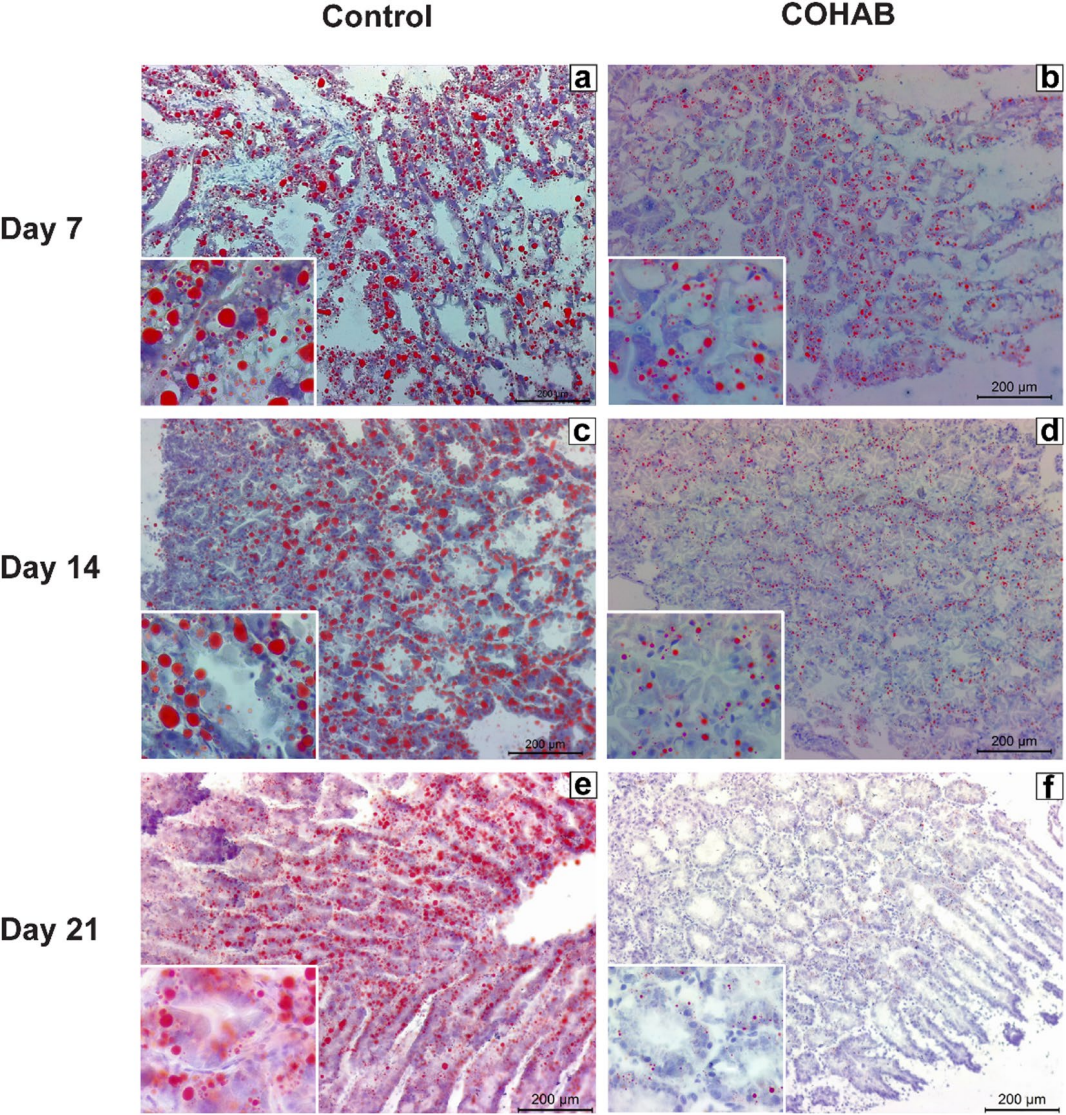
EHP infection reduces lipid droplet accumulation in the hepatopancreas (HP). Representative micrographs of Oil Red O stained hepatopancreatic tissue from control and COHAB tanks after 7, 14, and 21 days of cultivation. The red area indicates lipid droplets.

**Fig. 5:**
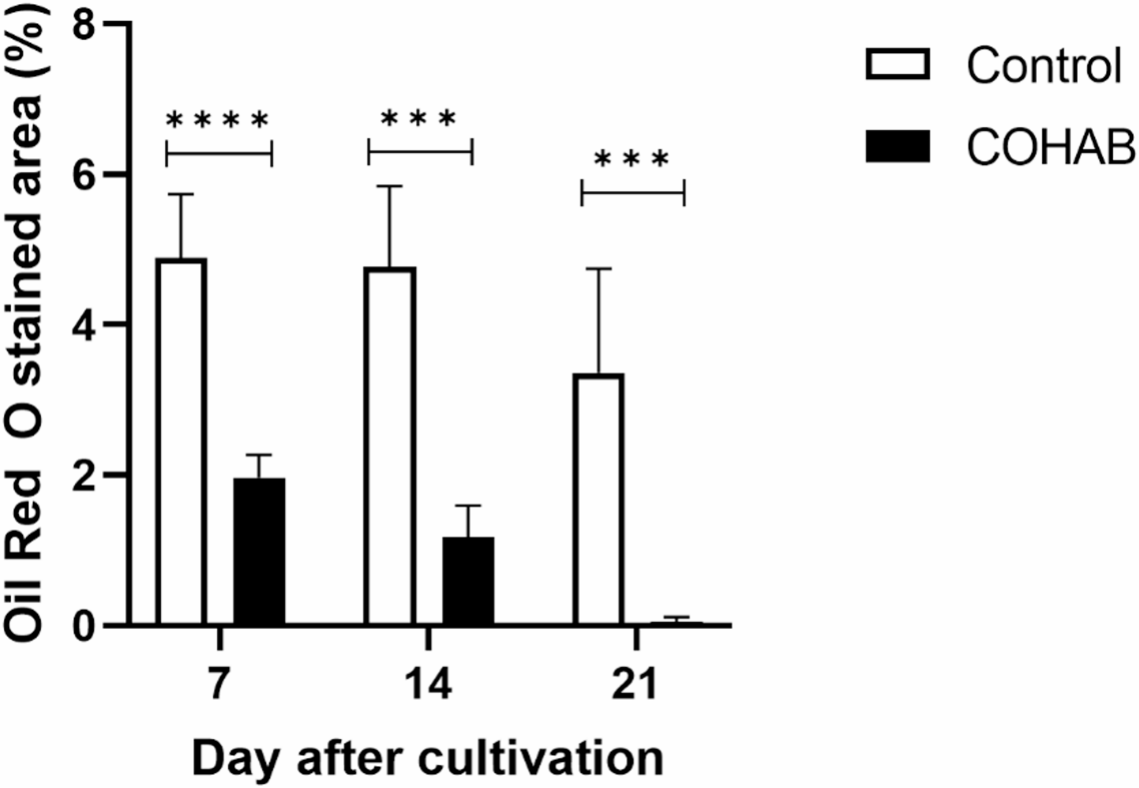
Lipid droplet accumulation in the hepatopancreas significantly decreased after EHP infection. The graph shows the percentage of Oil Red O-stained area in the hepatopancreas (HP) of shrimp from control (no-filled bar) and COHAB (EHP-infected, dark-filled bar) tanks after 7, 14, and 21 days of cultivation. Lipid droplet accumulation in the HP of infected shrimp significantly decreased over time compared to control shrimp. Data are presented as mean ± SD. The asterisks indicate statistical differences between control and COHAB groups at the same time point, tested by t-test. *** = *p* < 0.001, **** = *p* < 0.0001.

The results showed that lipid droplets were found in both B-cells and R-cells of the HP (Fig. 4). Interestingly, lipid accumulation in the HP of COHAB shrimp was significantly decreased when compared to the control group at all observed time points (Figs. 4 and 5). These results suggest that EHP infection impedes lipid accumulation in the HP from the early stages and exerts a chronic effect during long-term infection.

To further investigate the pathological impact of EHP infection on HP tissue structure, hematoxylin and eosin (H&E) staining was performed. On day 7, the HP from both groups showed similar characteristics; the HP tubules remained intact, and numerous B-cells were observed (Supplementary Figure S6). On day 14, shrimp from the COHAB tank showed a decrease in B cells and exhibited epithelial atrophy, with flattened epithelium (Supplementary Figure S7). In contrast, the control group maintained a normal tubular structure with abundant B-cells. On day 21, R-cells were observed in the control group but not in shrimp from the COHAB tank. In addition, shrimp from the COHAB tank exhibited fewer B-cells and smaller vacuoles within B-cells, along with flattened epithelial cells lining the HP tubules, compared to the control (Supplementary Figure S8).

Although EHP infection in the COHAB group was not confirmed directly in these specific samples, see materials and methods for rationale, it was verified in parallel samples from the same experimental group using nested PCR (Supplementary Figures S1−2 and S4-5). These results indicate progressive degeneration of the HP epithelium in shrimp exposed to EHP through cohabitation, which may reflect impaired digestive and metabolic function associated with EHP-induced tissue damage.

### EHP infection differentially alters expression of lipid droplet breakdown and lipid oxidation genes

**Fig. 6:**
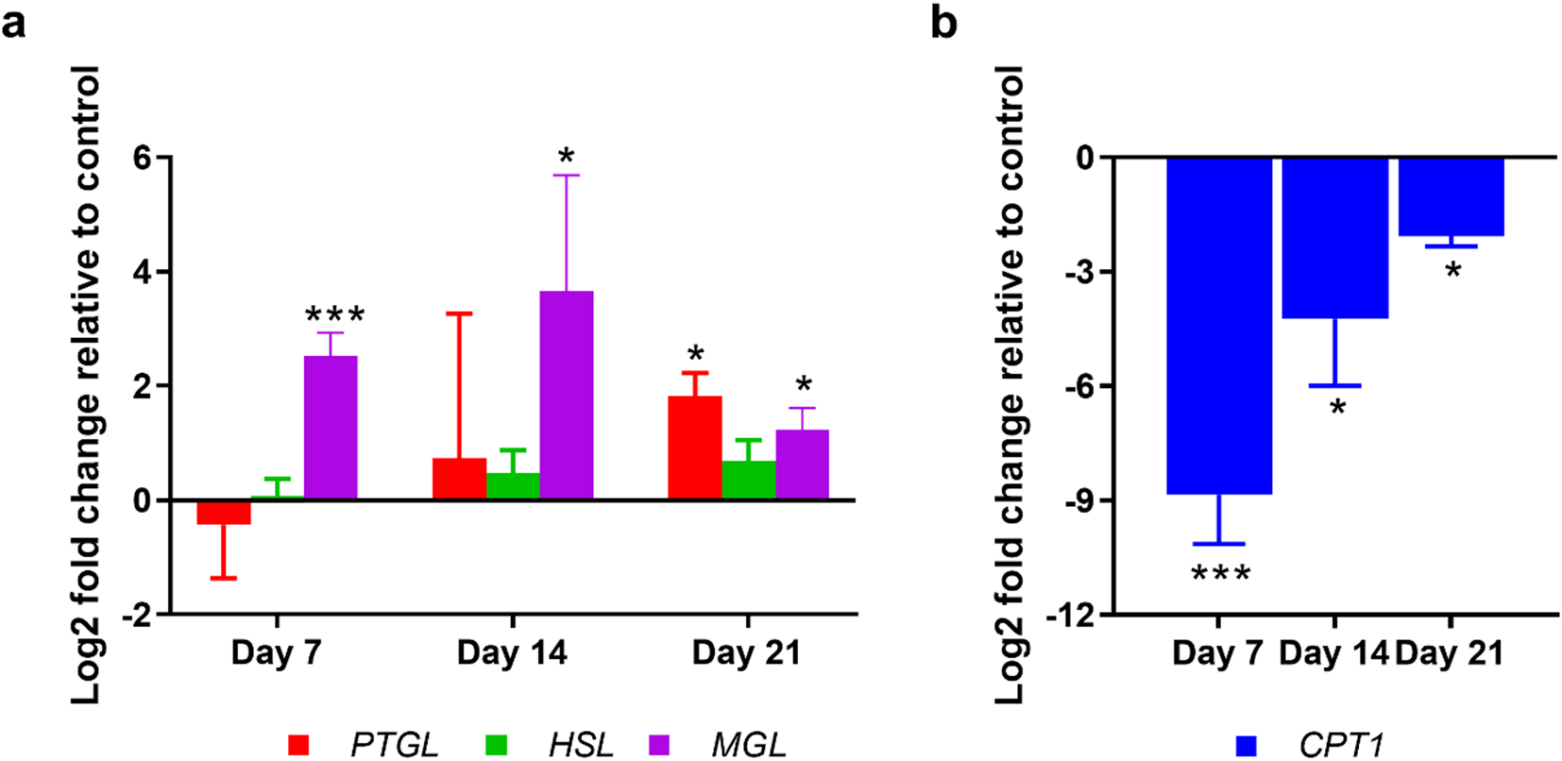
EHP infection alters expression of genes involved in lipid and lipid droplet breakdown. The bar graph shows log2-fold changes in mRNA expression of (**a**) lipid droplet breakdown (*PTGL*, *HSL*, *MGL*) and (**b**) lipid breakdown or fatty acid oxidation (*CPT1*) in the hepatopancreas of EHP-infected (COHAB) shrimp after 7, 14, and 21 days of cohabitation, relative to control shrimp at the same time points. *PTGL* = Pancreatic triacylglycerol lipase, *HSL* = Hormone-sensitive lipase, *MGL* = Monoacylglycerol lipase, *CPT1* = Carnitine palmitoyltransferase. Data are presented as mean ± SD. The asterisks denote statistical significance relative to the control group at the same time point, tested by t-test; * = *p* < 0.05, *** = *p* < 0.001.

Given the observed dramatic decrease in lipid droplets after 21 days of EHP infection, we further hypothesized that genes involved in lipid droplet breakdown would be upregulated following infection. To test this hypothesis, we examined the mRNA expression levels of key genes related to lipid droplet breakdown, including pancreatic triacylglycerol lipase (*PTGL*), hormone-sensitive lipase (*HSL*), and monoacylglycerol lipase (*MGL*) in the HP of EHP-infected shrimp over 21 days. The results showed that *PTGL* expression significantly increased only at 21 days, whereas *HSL* exhibited minimal changes across all time points (Fig. 6a). In contrast, *MGL* displayed significant increases at all three time points, with the highest expression observed on day 14.

Apart from lipid droplet breakdown, we further hypothesized that EHP infection would increase fatty acid oxidation to counteract its inability to synthesize ATP. The mRNA expression level of the gene involved in fatty acid oxidation, Carnitine palmitoyltransferase 1 (*CPT1*), was examined. The result showed that *CPT1* expression was significantly downregulated in EHP-infected shrimp compared to the control group at all time points examined (Fig. 6b). The most pronounced downregulation was observed on day 7. Interestingly, while *CPT1* expression remained lower than control levels, the extent of downregulation decreased over time, suggesting a progressive increase in lipid oxidation during the later stages of infection.

### EHP infection induced time-dependent changes in the expression of genes related to lipid transport and synthesis

**Fig. 7:**
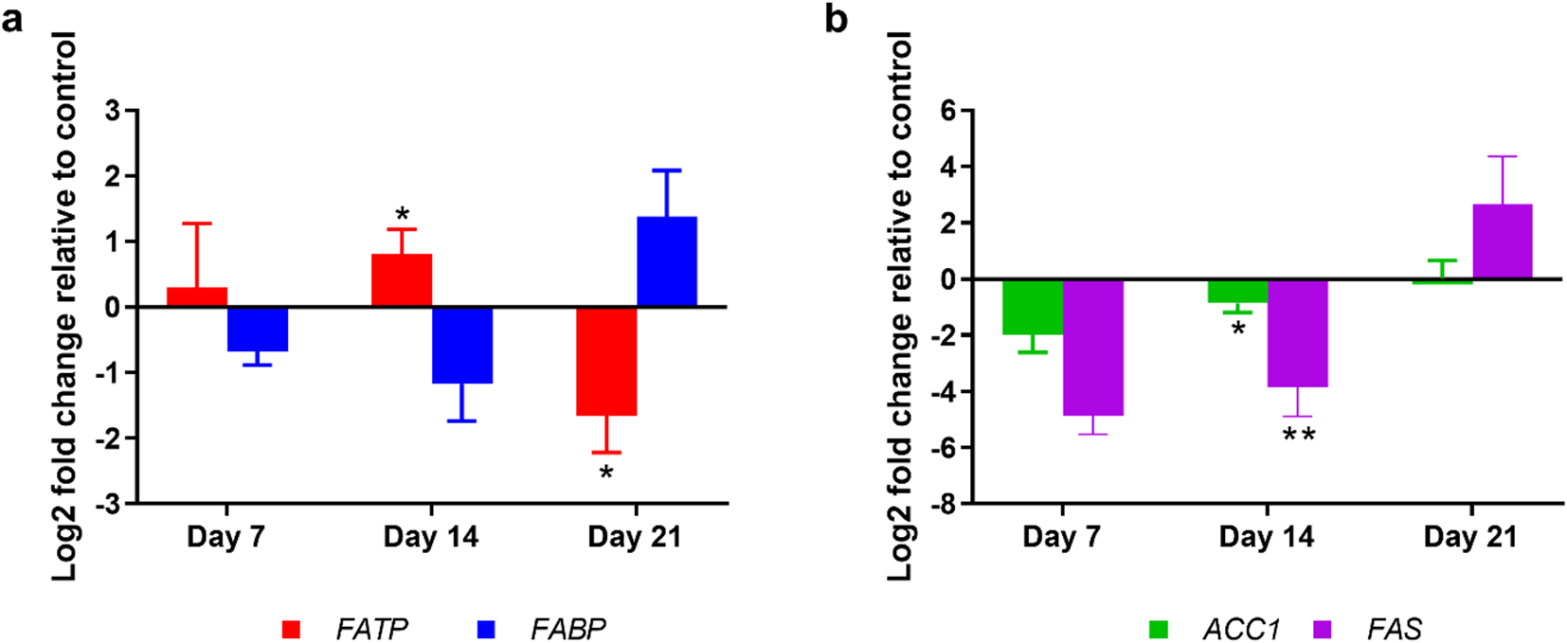
EHP infection induces time-dependent changes in lipid transport and synthesis-related gene expression. The graph shows log2-fold changes in mRNA expression of (**a**) lipid transport genes (*FABP*,* FATP*) and (**b**) lipid synthesis genes (*ACC1*, *FAS*) in the hepatopancreas of EHP-infected shrimp after 7, 14, and 21 days of cohabitation, relative to control shrimp. *FABP* = fatty acid-binding protein, *FATP* = fatty acid-transport protein, *ACC1* = Acetyl-CoA carboxylase 1, *FAS* = fatty acid synthase. Data are presented as mean ± SD. The asterisks denote statistical significance relative to the control group at the same time point, tested by t-test; * = *p* < 0.05, ** = *p* < 0.01

The steady state of lipid level or lipid droplet depends on the interplay between synthesis and breakdown. To investigate the impact of EHP infection on lipid synthesis, we examined the expression of key genes involved in fatty acid transport, and synthesis in the HP of infected shrimp over 21 days (Fig. 7). The results revealed a complex and time-dependent regulation of these genes.

We first examined genes involved in lipid transport, which exhibited distinct temporal patterns (Fig. 7a). Fatty acid-transport protein (*FATP*), which uptakes fatty acids into cells, displayed a slight increase at day 7, followed by significant upregulation at day 14, and then a marked downregulation by day 21. This pattern suggests an initial enhancement of fatty acid uptake capacity during the early stages of infection, followed by a sharp decline, which may contribute to the observed lipid droplet accumulation. In contrast, fatty acid-binding protein (*FABP*) expression increased at day 21, diverging from the uptake pattern. Although not statistically significant, this may suggest enhanced intracellular lipid distribution as infection progresses.

Next, we analyzed genes involved in de novo lipid synthesis, which showed consistent initial suppression followed by divergent responses (Fig. 7b). Both Acetyl-CoA carboxylase 1 (*ACC1*) and fatty acid synthase (*FAS*) showed non-significant downregulation at day 7, progressing to significant downregulation at day 14. However, by day 21, their expressions diverged: Acc1 returned to near-control levels, while *FAS* shifted to non-significant upregulation. This pattern indicates a strong suppression of lipid synthesis during 14 days of infection, with a potential recovery or compensatory response beginning by day 21.

These temporal changes in gene expression reveal a complex metabolic adaptation to EHP infection. The early stages (days 7–14) exhibit an enhanced lipid uptake capacity coupled with suppressed synthesis. The later stage (day 21) shows a more varied response, potentially reflecting the host’s attempt to restore metabolic homeostasis as the infection progresses.

## Discussion

### Impaired digestive enzyme activity and growth retardation

Our findings reveal that the adverse effects of EHP on digestive enzymes emerge progressively rather than immediately after infection. In the early stage of infection (day 7), there was no difference in either enzyme activity or mRNA expression levels of the digestive enzymes compared to those of the control (Figs. 2 and 3). However, by day 14 and 21, a significant reduction in both enzyme activity and mRNA expression was observed (Figs. 2 and 3). Our result aligns with a transcriptomic study that mRNA expression levels of trypsin, amylase and lipase were reduced after EHP infection^22^.

Our findings on enzyme activity variations mainly support prior studies, with some notable difference in lipase activity. A previous study also found that amylase activity in the hepatopancreas (HP) was significantly reduced after an EHP challenge, starting from 15 days post-challenge and continuing through 30, 60, and 90 days^28^. However, we observed a nonsignificant reduction in lipase activity in the HP which did not correspond with the previous one, where lipase activity in the HP was significantly decreased after EHP infection^28^. The discrepancy could be attributed to differences in infection intensity, experimental conditions, or host nutritional status. Nonetheless, our results collectively suggest that EHP infection impairs the shrimp’s ability to digest dietary nutrients effectively, thereby reducing available nutrients and ultimately contributing to the stunted growth commonly observed in EHP-infected shrimp.

### Lipid droplets and metabolic imbalance

Lipid droplets serve as critical energy reservoirs, enabling organisms to regulate energy balance under fluctuating environmental conditions^31^. Various pathogens exploit or manipulate these reservoirs to favor their own survival and replication^32^. In our study, EHP infection caused a continuous reduction of lipid accumulation in the HP from the early stages of infection. Although lipid droplets were depleted at all observed time points, the expression patterns of lipid metabolism-related genes varied across stages.

In the early stages (day 7 and day 14), lipid droplet accumulation in the HP was significantly lower in EHP-infected shrimp compared to control shrimp (Fig. 4). The depletion together with the upregulation of *MGL* (Fig. 6a), a lipid droplet breakdown enzyme, suggest that increased lipid breakdown contributed to reduced lipid storage. Meanwhile, both fatty acid oxidation (*CPT1*) and lipid synthesis (*ACC1* and *FAS*) were strongly suppressed in this phase, (Figs. 6b and 7b), whereas fatty acid transport capacity (*FATP*) was significantly upregulated at day 14 (Fig. 7a). These results suggest a metabolic imbalance where lipid breakdown outpaces utilization (uptake and oxidation) and replenishment (synthesis). The continued downregulation of lipid synthesis from day 7 might be a strategy of shrimp to conserve energy and maintain homeostasis or a manipulation of EHP to secure its energy needs.

At the late phase, lipid droplet levels drop significantly below those of naïve shrimp, corresponding to the upregulation of both *PTGL* and *MGL*, which enhances lipid droplet breakdown (Figs. 5 and 6a). Fatty acid oxidation (*CPT1*) remains downregulated, albeit to a lesser extent than in earlier stages (Fig. 6b**)**. Concurrently, there is a partial recovery of lipid synthesis, as evidenced by *ACC1* expression returning to levels comparable to the control group and *FAS* being slightly upregulated (Fig. 7b). The upregulation of lipid synthesis genes suggests an attempt by the shrimp to compensate for the escalation of lipid breakdown (both lipid oxidation and lipid droplet breakdown). However, the rate at which lipid synthesis tries to recover cannot negate the rate of breakdown, resulting in a net loss of lipid droplets. Notably, although lipid oxidation remained downregulated in EHP-infected shrimp compared to the control, it showed an increase relative to the infected shrimp in earlier stages.

Overall, these results show that EHP infection consistently reduces lipid droplet accumulation as well as altering lipid metabolic pathways, leading to depletion of lipid storage. This effect of EHP on lipid reservoir underscores the chronic nature of EHP infection which, while not acutely lethal, exerts ongoing effects that eventually stunt shrimp growth and productivity.

Our findings are consistent with previous research showing that EHP infection significantly alters shrimp lipid metabolism by downregulating fatty acid elongation and degradation, unsaturated fatty acid biosynthesis, and juvenile hormone degradation^29^. Furthermore, alterations in lipid metabolism are also observed during viral infection. For instance, white spot syndrome virus (WSSV) infection induces both lipolysis and lipogenesis at different stages to support virus replication and morphogenesis, with a shift in lipid droplet distribution and fatty acid utilization^36^. Taken together, these observations highlight the significant impact of both parasitic and viral infections on shrimp lipid metabolism, suggesting that strategies aimed at maintaining lipid homeostasis could improve resilience to a range of pathogens.

## Applications and management strategies

The economic importance of *P. vannamei* aquaculture underscores the need for effective strategies to manage EHP outbreaks. Since we found that EHP infection depletes lipid reserves and disrupts lipid metabolism, dietary lipid supplementation may help mitigate these effects.

A previous study showed that adding linolenic acid (C18:3n-3), a polyunsaturated fatty acid found in plant seeds and oils, at 2.4 g/kg diet improved growth, non-specific immunity, and antioxidant activity in EHP-infected shrimp^37^. Additionally, a previous study found that shrimp fed a diet containing 11% lipid and 1.8% lecithin (widely used as phospholipids supplements in aquafeed) displayed optimal growth, hemolymph parameters, body texture, immune responses, and notably the highest mRNA expression of hepatopancreatic lipase, compared to shrimp receiving lower lipid and lecithin levels^38^.

These findings together with our results suggest that lipid supplementation could help counteract the negative impacts of EHP infection. By combining leveraging linolenic acid, lecithin, and other dietary approaches with optimal environmental management and early EHP detection, shrimp farms may improve shrimp health and resilience against EHP outbreaks.

## Conclusion

Overall, our results elucidate a crucial link between EHP infection, lipid droplet accumulation, and digestive enzyme activity in the shrimp HP. Our findings demonstrate the long-term, cumulative effects of impaired nutrient acquisition coupled with progressively increased lipid breakdown in EHP-infected shrimp. By demonstrating how EHP disrupts both lipid synthesis and breakdown, as well as digestive enzyme function, we have clarified the underlying molecular mechanism that is potentially associated with observed growth retardation caused by EHP. These insights not only advance our fundamental understanding of host-pathogen interactions in shrimp but also offer potential approaches for combating EHP infection. Further studies examining the molecular details of how dietary lipid supplementation affects EHP-infected shrimp will be valuable next steps.

## Materials and methods

### Shrimp cultivation and sample collection

To acquire shrimp infected with EHP, a cohabitation assay was established following a prior study^39^. Two containers (assigned as COHAB tanks) containing approximately 300 L of artificial seawater at 20 ppt were set up, each with ten naturally EHP-infected juvenile shrimp kept inside a cage and 100 naïve juvenile shrimp (2–3 g) that freely swam in the tank. Water was cycled between the two tanks throughout the experiment. Five shrimp from the same batch as the donor EHP-infected shrimp were tested by nested PCR to confirm EHP infection prior to initiating the cohabitation experiment (Supplementary Figure S9). In addition, two containers (assigned as control tanks) containing 100 naïve shrimp were set up as a control experiment, with water also cycled between these two tanks for the duration of the experiment. During the experiment, the following factors were measured and controlled: pH of water = 8, dissolved oxygen level > 4.5 mg/L, nitrate level < 0.1 mg/L, and total ammonia < 5 mg/L. Since the molting stage has been shown to influence the expression of digestive enzymes, shrimp in the inter-molt stage were identified based on a prior study^13^ and were collected from both experiments on days 7, 14, and 21 after the start of cultivation. The shrimp were collected in four sets for the examination of (A) digestive enzyme activity, (B) lipid droplet accumulation in the hepatopancreas (HP), (C) Hematoxylin and Eosin (H&E) staining, and (D) mRNA expression of digestive enzymes and lipid metabolism-related genes.

The animal experiments were conducted in accordance with the guidelines for the care and use of animals in scientific research. The experimental protocol was approved by the Institutional Care and Use Committee at the Faculty of Science, Mahidol University, under protocol number MUSC64-035-584.

### Determination of digestive enzyme activity

To examine digestive enzyme activity, nine shrimp from control and cohabitation tanks at each time point were dissected to collect stomach and HP. Three of each tissue type were pooled into one sample (for a total of three pooled samples per time point). The tissues were ground in 50 mM Tris-HCl buffer (pH 8) containing 200 mM NaCl. For the HP, half of the sample was aliquoted for DNA extraction and EHP verification prior to the next step. Since EHP has been shown to not distribute uniformly throughout the HP^40^, the HP samples subjected to nested PCR had to be ground to ensure homogeneity of EHP distribution prior to being used as a template in the PCR. The sample was then centrifuged at 10,000 g, 4 °C for 30 min. The supernatant was collected and stored at −80 °C until used for protein concentration and enzyme activity determination. Total protein concentration was measured by Bradford assay using bovine serum albumin (BSA) as a standard.

#### Trypsin activity

Trypsin activity was determined according to a previous study^41^ using Bensoyl-L-arginine-p-nitroanilide (BAPNA) as a substrate. One unit of trypsin activity corresponded to 1 µmol of 4-nitroaniline released per minute. Specific trypsin activity was defined as U/mg total protein.

#### Amylase activity

Amylase activity was estimated by a protocol adapted from a previous study^42^. One unit of amylase activity corresponded to 1 µmol of maltose released per minute. Specific amylase activity was defined as U/mg total protein.

#### Lipase activity

To test lipase activity, the protocol was modified from a previous study^43^. One unit of lipase activity corresponded to 1 µmol of β-napthol released per minute. Specific lipase activity was defined as U/mg total protein.

### DNA extraction and EHP verification

To the mixture that was aliquoted for DNA extraction, 180 µl of lysis buffer (2 M Tris pH 9.0, 0.5 M EDTA, 5 M NaCl, and 10% SDS) and 20 µl of 5 mg/ml proteinase K were added. The mixture was then incubated at 56 °C for 1 h before extracting total DNA using the QIAamp DNA Mini Kit (Qiagen, Germany) following the manufacturer’s protocol. EHP infection was detected using a nested PCR assay detecting a spore wall protein (SWP)-encoding gene as previously reported^44^. Primer sequences and the nested PCR cycling program used in this study were the same as those in the previous study. The PCR mixture of both steps contained 1X Green PCR Master Direct-Load (Biotechrabbit, Germany), 0.2 µM of forward and reverse primers (SWP_1F/SWP_1R for the first step and SWP_2F/SWP_2R for the second step), and water up to 12.5 µl. The templates for the first and second steps of nested PCR were 100 ng of hepatopancreatic DNA and 1 µl of the first step PCR product, respectively. A pGEM-SWP plasmid and a no-template reaction were used as positive and negative controls, respectively. Actin was used as an internal control, with the following primer sequences: forward 5′-GACTCGTACGTGGGCGACGAGG-3′ and reverse 5′-AGCAGCGGTGGTCATCTCCTGCTC-3′. For actin amplification, 100 ng of hepatopancreatic DNA served as the template. PCR results were visualized on a 1.5% agarose gel. Amplicon sizes of the first and second steps were 514 and 148 bp, respectively, while the actin was 550 bp. EHP infection was classified as follows: Heavy infection referred to samples that gave positive PCR products in both first and second steps of nested PCR, while light infection was defined as samples that exclusively gave a positive PCR product in the second PCR step. Uninfected samples were PCR-negative in both steps.

### Determination of mRNA expression of digestive enzymes and lipid metabolism-related genes

To determine the mRNA expression of digestive enzymes and genes involved in lipid metabolism in the HP, nine shrimp from control and cohabitation tanks at each time point were dissected to collect the HP. Three HP tissues were pooled into one sample (for a total of three pooled samples per time point). The tissue samples were ground in liquid nitrogen to powder and divided into two portions for DNA extraction and EHP verification as described earlier and RNA extraction.

### RNA extraction and complementary DNA (cDNA) synthesis

To extract RNA, 500 µl RiboZol RNA Extraction Reagent (VWR Life Science, USA) was added to the ground powder. The total RNA was then isolated according to the manufacturer’s protocol. Contaminating DNA in the extracted RNA samples was removed using RQ1 RNase-Free DNase (Promega, USA). The RNA concentration was determined using a Nanodrop spectrophotometer. The purity of the RNA was determined by the A260/A280 ratio, which ranged between 1.8 and 2.0. To synthesize cDNA, the ImProm-II™ Reverse Transcription System (Promega, USA) was used with 1 µg of total RNA and oligo-dT as a template and primers, respectively.

### Quantitative real-time PCR (qPCR) analysis

The mRNA expression levels of digestive enzymes and lipid metabolism-related genes in cDNA samples were determined using qPCR with primers shown in Table 1. The qPCR reaction consisted of 5 ng of cDNA, 1X KAPA SYBR^®^ FAST qPCR Master Mix (Roche, Switzerland), 0.2 µM of each forward and reverse primer, and water up to 20 µl. The qPCR was performed using the CFX Connect™ Real-Time System (Bio-Rad, USA) with a two-step procedure: (1) initial denaturation at 95 °C for 3 min, (2) 40 cycles of denaturation at 95 °C for 10 s and annealing at 59 °C for 30 s, followed by melt curve analysis to confirm specificity of the qPCR product. The qPCR reactions were performed in triplicate for each sample. Elongation Factor-1 alpha (EF-1α) was used as a reference gene. In cases where samples yielded a detectable Ct value for EF-1α but an undetectable Ct value for the target gene, the missing values for the target gene were imputed using the R package ‘nondetects’^45^. Subsequently, the relative expression levels of the genes of interest were calculated using the Pfaffl method^46^ to determine the fold change in mRNA abundance following EHP infection. These changes were then transformed into a log2-fold change. Bar graphs and t-test analyses were generated using GraphPad Prism version 9.0.

**Table 1:** Primers used in this study.

Gene	Primer name	Primer sequence (5’ ◊ 3’)	Reference
Elongation factor-1 alpha (*EF-1α*)	EF1-F	TTCCGACTCCAAGAACGACC	^48^
	EF1-R	GAGCAGTGTGGCAATCAAGC	
Trypsin	Tryp-qF1	ACGATTTGGTCTCCACGGTC	This study
	Tryp-qR1	CGTAGATGTCCCTGCACTCC	
Amylase	Amy-qF2	ACCAAGGCACTGACTACGTG	This study
	Amy-qR2	AGATGAAAGGTCTGGCACCG	
Lipase	Lip1-F	GCTGGCTTACAAACTCGC	^29^
	Lip1-R	AATCAATGCTCGCAGGAA	
Pancreatic triacylglycerol lipase (*PTGL*)	PTGL-qF	GGGACTTCCTCCTTTGGACG	This study
	PTGL-qR	ACGTAGGTCTTGGTCGGGTA	
Hormone-sensitive lipase (*HSL*)	HSL-qF	CCCTCAATGTCCCAGTCACC	This study
	HSL-qR	TTCCGTCAACCCAAGCAGTT	
Monoacylglycerol lipase (*MGL*)	MGL-qF	TTCGTGTCAGTGAGAACGGG	This study
	MGL-qR	AAGGCGATTCGAGCACAAGA	
Acetyl-CoA carboxylase 1 (*ACC1*)	Acc1-F	TGCATAGAAACGGCATTGCG	^49^
	Acc1-R	TTTGACACCTGAGCCAGACC	
Carnitine palmitoyl transferase 1 (*CPT1*)	Cpt1-F	ACTCCCGATAAGCACACC	^49^
	Cpt1-R	TTCATACATCCACCCCCT	
Fatty acid-binding protein (*FABP*)	Fabp-F	CGACCACCACTTTCAAGACC	^49^
	Fabp-R	TAGCATCTTGTCGTCGGTGA	
Fatty acid-transport proteins (*FATP*)	Fatp-F	TTCCAGGGGTGTCTTAGCTG	^49^
	Fatp-R	GTACAGGTAGCGGCAGATCT	
Fatty acid synthase (*FAS*)	FAS-f-F	CAGGTGGAGATGCTCCTCGTGTT	^50^
	FAS-f-R	GGTGACTAGCTCGGCTACATGGTT	

### Cryosectioning and lipid droplets staining

To preserve shrimp tissue for cryosectioning, shrimp were fixed overnight in 4% paraformaldehyde at 4 °C. The shrimp were then rinsed in phosphate-buffered saline (PBS). Subsequently, they were transferred to a gradient sucrose solution series with overnight incubation at 4 °C for each step, beginning with 10%, followed by 20%, and finally 30%. The HP of the preserved shrimp was dissected, embedded into Optimal Cutting Temperature (OCT) compound, and cryopreserved using liquid nitrogen. The embedded tissue was then sliced into 10-µm-thick sections using a cryostat set at −25 °C.

To stain lipid droplets, the tissue sections were first placed in propylene glycol for 5 min and then incubated with the Oil Red O staining solution (Abcam, USA) at 60 °C for 30 min. Afterward, the sections were immersed in 85% propylene glycol for 1 min and rinsed with distilled water three times. Subsequently, the sections were counterstained with hematoxylin for 1 min and thoroughly rinsed once in tap water and twice in distilled water. Finally, the sections were mounted with glycerol, covered with a coverslip, and observed under a light microscope.

### Determination of lipid droplets accumulation in the HP

The accumulation of lipid droplets in the HP was determined by observing Oil Red O-stained HP sections under a light microscope. Five micrographs of HP from two groups, ‘Control’ and ‘COHAB,’ at each time point were analyzed for the area of Oil Red O staining using ImageJ software^47^. The average stained area from the five micrographs was calculated. Statistical analysis was conducted using GraphPad Prism 9.0, employing a t-test comparing Control and COHAB groups at the same time point.

### Histological study of shrimp hepatopancreas

To examine how EHP affects shrimp HP, shrimp from both the control and COHAB groups, collected at each time point, were injected with Davidson’s fixative and immersed in it for 24 h. The fixed shrimp were then preserved in 70% ethanol prior to processing for paraffin embedding using a tissue processor. Shrimp cephalothorax tissues were sectioned into 4–5 μm thick sections and stained with Hematoxylin and Eosin (H&E). The stained sections were then observed under a light microscope.

## Supplementary Information

Below is the link to the electronic supplementary material.

Supplementary Material 1

## Data Availability

All data generated or analyzed during this study are included in this published article and its Supplementary Information file.
