# Supplementary material for: Ecytonucleospora hepatopenaei causes lipid droplet depletion and imbalanced lipid metabolism in Penaeus vannamei
Source: Sci Rep. 2025 Oct 24;15:37198. doi: 10.1038/s41598-025-21037-y (PMC12552485; doi:10.1038/s41598-025-21037-y)
Supplement: Supplementary file 1 — Supplementary Material 1 [file 41598_2025_21037_MOESM1_ESM.pdf]

Ecytonucleospora hepatopenaei Causes Lipid Droplet Depletion and Imbalanced Lipid Metabolism in *Penaeus vannamei*

Satika Yuanlae[1,2,#], Dararat Thaiue[3,4,#], Sukanya Saedan[2,5], Kamonluk Kittiwongpukdee[2,5], Rapeepun Vanichviriyakit[2,5], Niti Chuchird[4,*], Ornchuma Itsathitphaisarn[1,2,**]

[1]Department of Biochemistry, Faculty of Science, Mahidol University, Rama VI Rd., Bangkok 10400, Thailand.

[2]Center for Excellence in Shrimp Molecular Biology and Biotechnology (Centex Shrimp), Faculty of Science, Mahidol University, Rama VI Rd., Bangkok 10400, Thailand.

[3]Aquatic Animal Health Research Team (AQHT), Integrative Aquaculture Biotechnology Research Group, National Center for Genetic Engineering and Biotechnology (BIOTEC), National Science and Technology Development Agency (NSTDA), Yothi office, Rama VI Rd., Bangkok 10400, Thailand.

[4]Department of Fishery Biology, Faculty of Fisheries, Kasetsart University, 50 Ngamwongwan Rd., Chatuchak, Bangkok, 10900, Thailand.

[5]Department of Anatomy, Faculty of Science, Mahidol University, Rama VI Rd., Bangkok 10400, Thailand.

#These authors contribute equally in this manuscript

*Co-corresponding author

Email: ffisntc@ku.ac.th

**Corresponding author

Email: ornchuma.its@mahidol.edu

Supplementary information

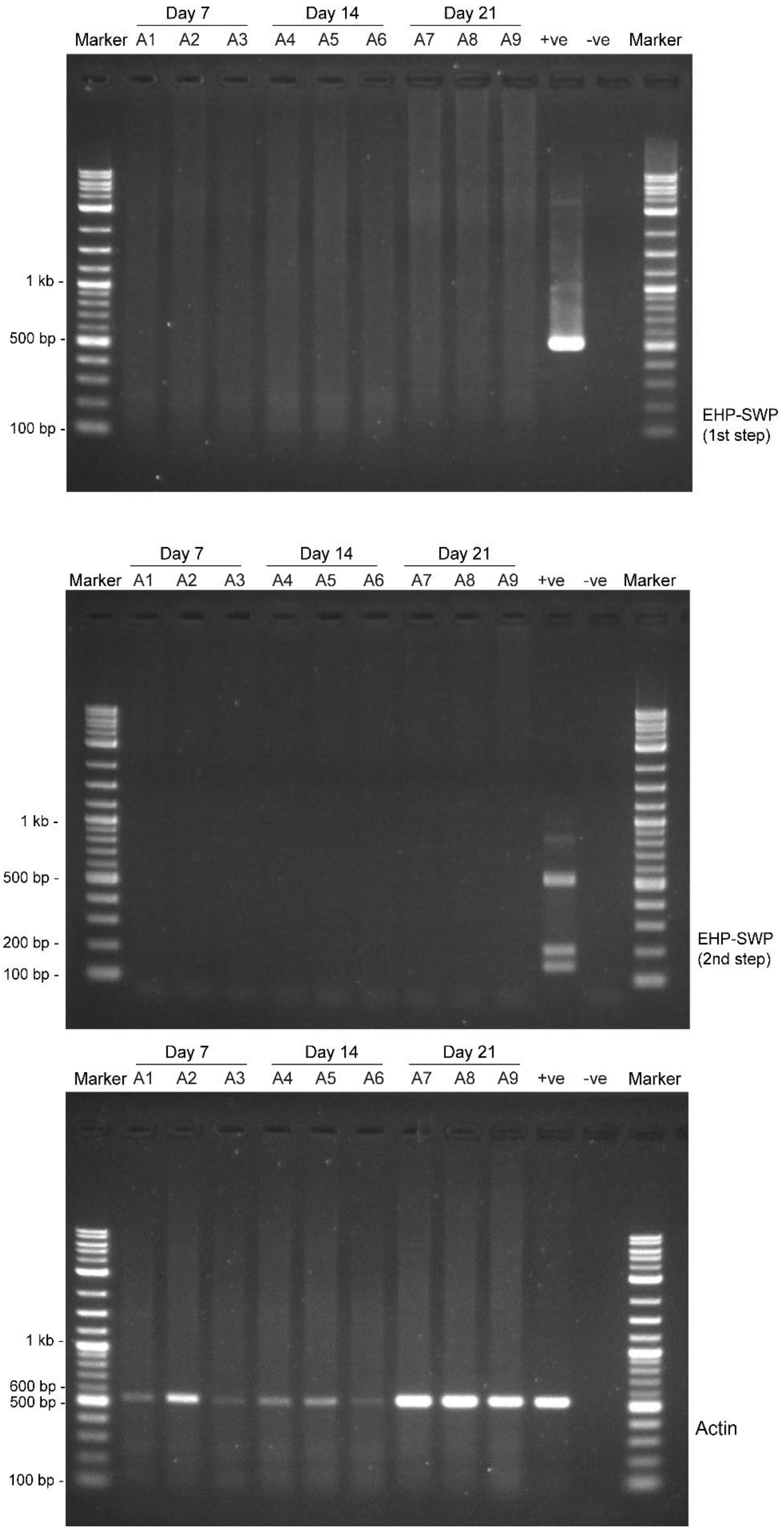

Supplementary Figure S1 Nested PCR detecting the SWP gene in shrimp from control tank. At 7, 14, and 21 days after the culture, shrimp collected for the determination of digestive enzyme activity (A1-9) were tested for EHP infection. Amplicon size of 1st step (top panel), 2nd step (middle panel), and actin (bottom panel) amplification are 514, 148, and 550 bp, respectively. +ve; positive control. –ve; negative control.

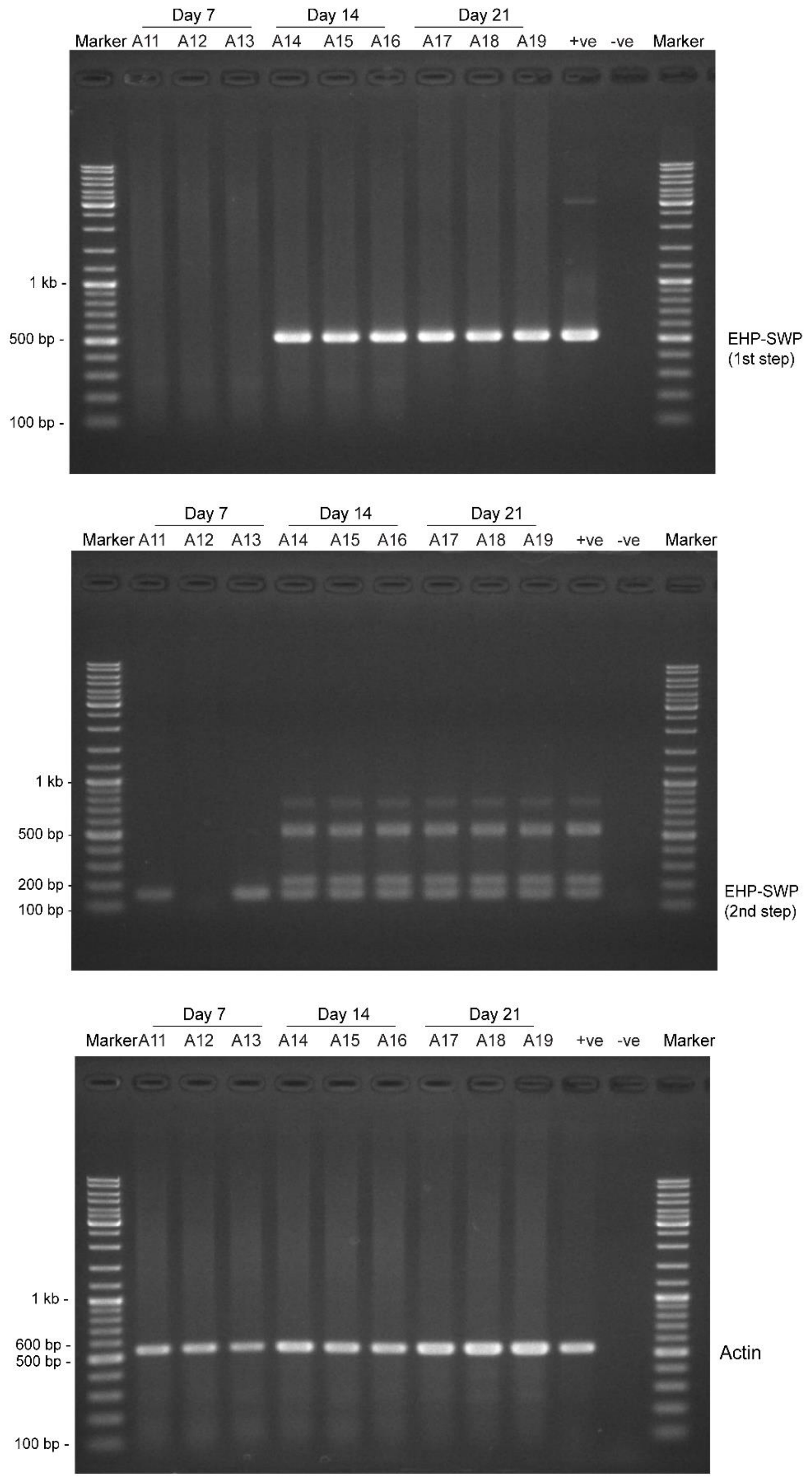

Supplementary Figure S2 Nested PCR detecting the SWP gene in shrimp from COHAB tank. At 7, 14, and 21 days after the culture, shrimp collected for the determination of digestive enzyme activity (A11-19) were tested for EHP infection. Amplicon size of 1st step (top panel), 2nd step (middle panel), and actin (bottom panel) amplification are 514, 148, and 550 bp, respectively. +ve; positive control. –ve; negative control.

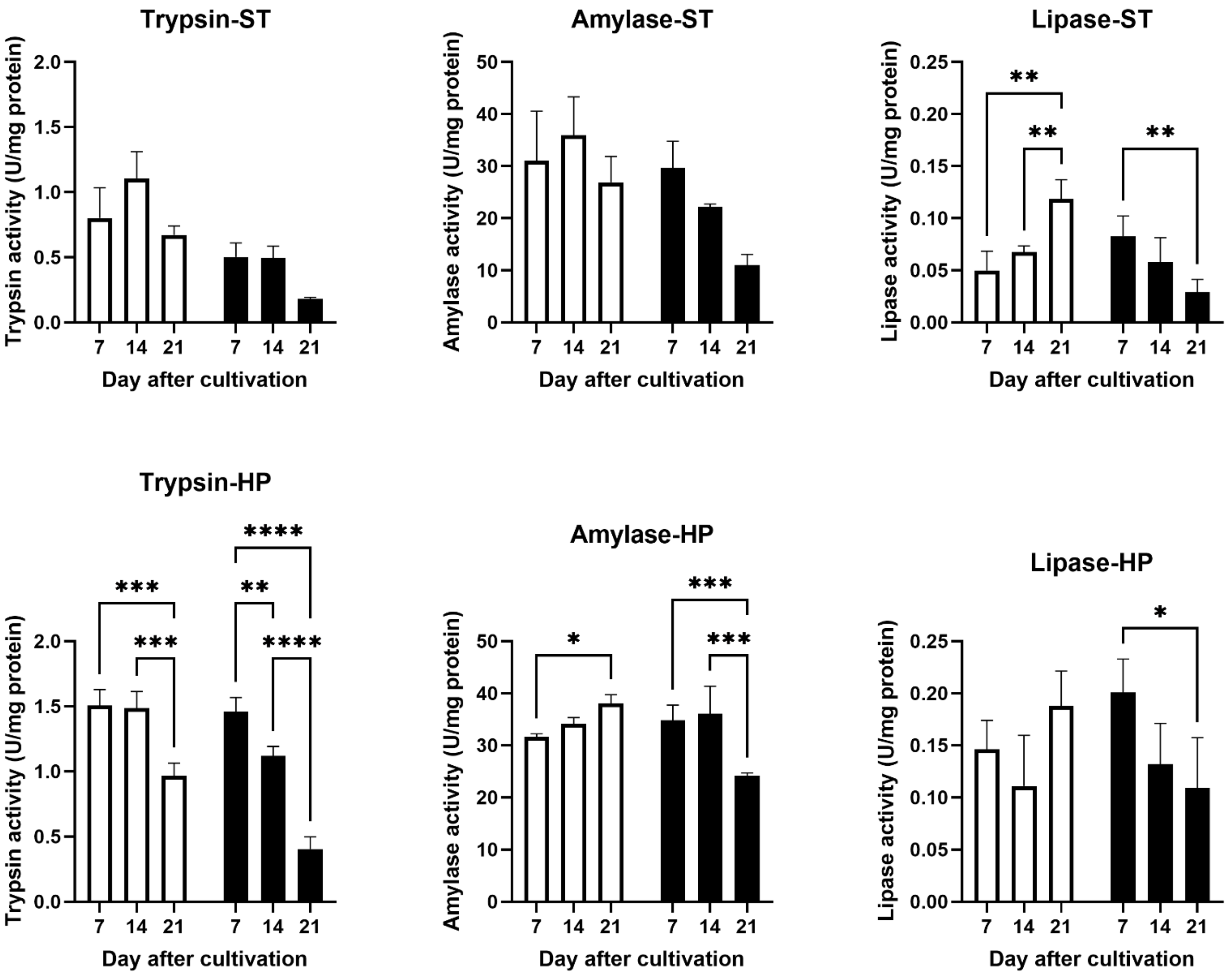

Supplementary Figure S3 Simple effects of time on digestive enzyme activity within each group. Bar graphs show trypsin, amylase, and lipase activities in the hepatopancreas (HP) and the stomach (ST) of shrimp from control (uninfected, open bars) and COHAB (EHP-infected, filled bars) tanks at 7, 14, and 21 days after cultivation. Analyses were performed by Tukey's multiple comparisons test following two-way ANOVA. Simple effects of time were examined only where a significant interaction was detected; thus, trypsin and amylase in the ST were not tested. Significant differences between time points within each group are indicated: * = $p < 0.05$, ** = $p < 0.01$, *** = $p < 0.001$, **** = $p < 0.0001$. Enzyme activity is expressed as U/mg protein.

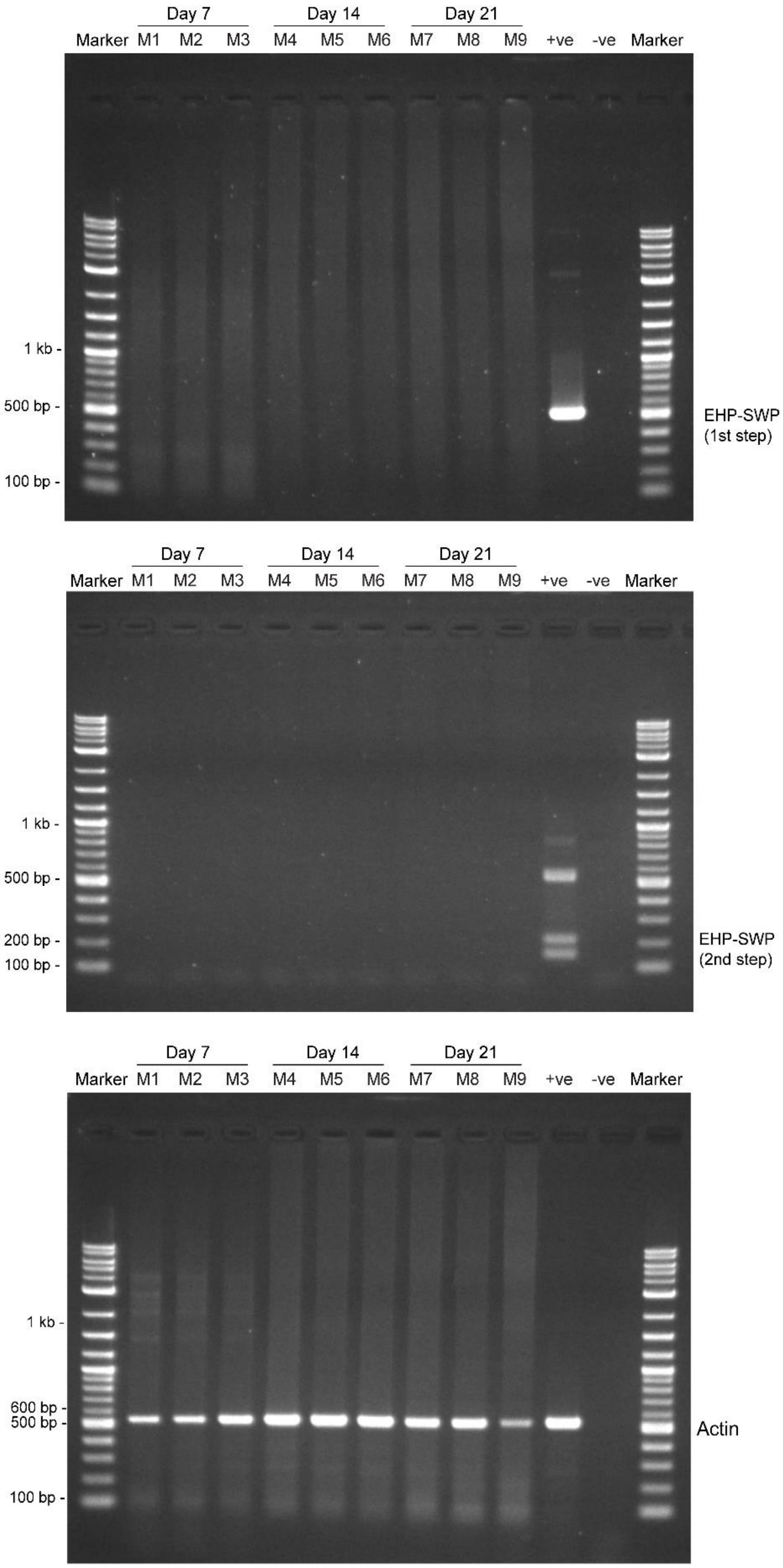

Supplementary Figure S4 Nested PCR detecting the SWP gene in shrimp from control tank. At 7, 14, and 21 days after the culture, shrimp collected for the determination of mRNA expression of digestive enzyme-encoding genes and lipid metabolism-related genes (M1-9) were tested for EHP infection. Amplicon size of 1st step (top panel), 2nd step (middle panel) and actin (bottom panel) amplification are 514, 148, and 550 bp, respectively. +ve; positive control. –ve; negative control.

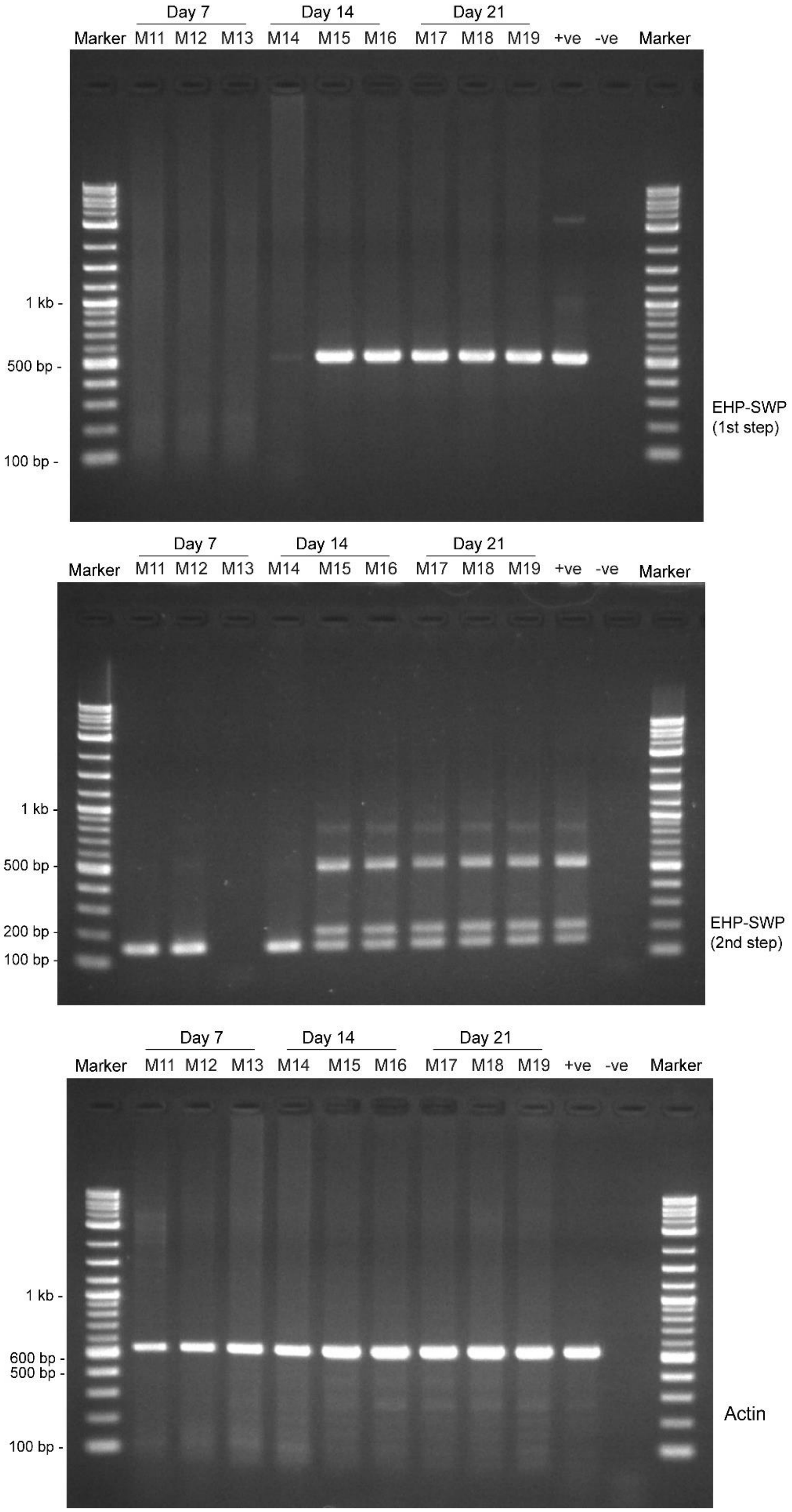

Supplementary Figure S5 Nested PCR detecting the SWP gene in shrimp from COHAB tank. At 7, 14, and 21 days after the culture, shrimp collected for the determination of mRNA expression of digestive enzyme-encoding genes and lipid metabolism-related genes (M11-19) were tested for EHP infection. Amplicon size of 1st step (top panel), 2nd step (middle panel), and actin (bottom panel) amplification are 514, 148, and 550 bp, respectively. +ve; positive control. –ve; negative control.

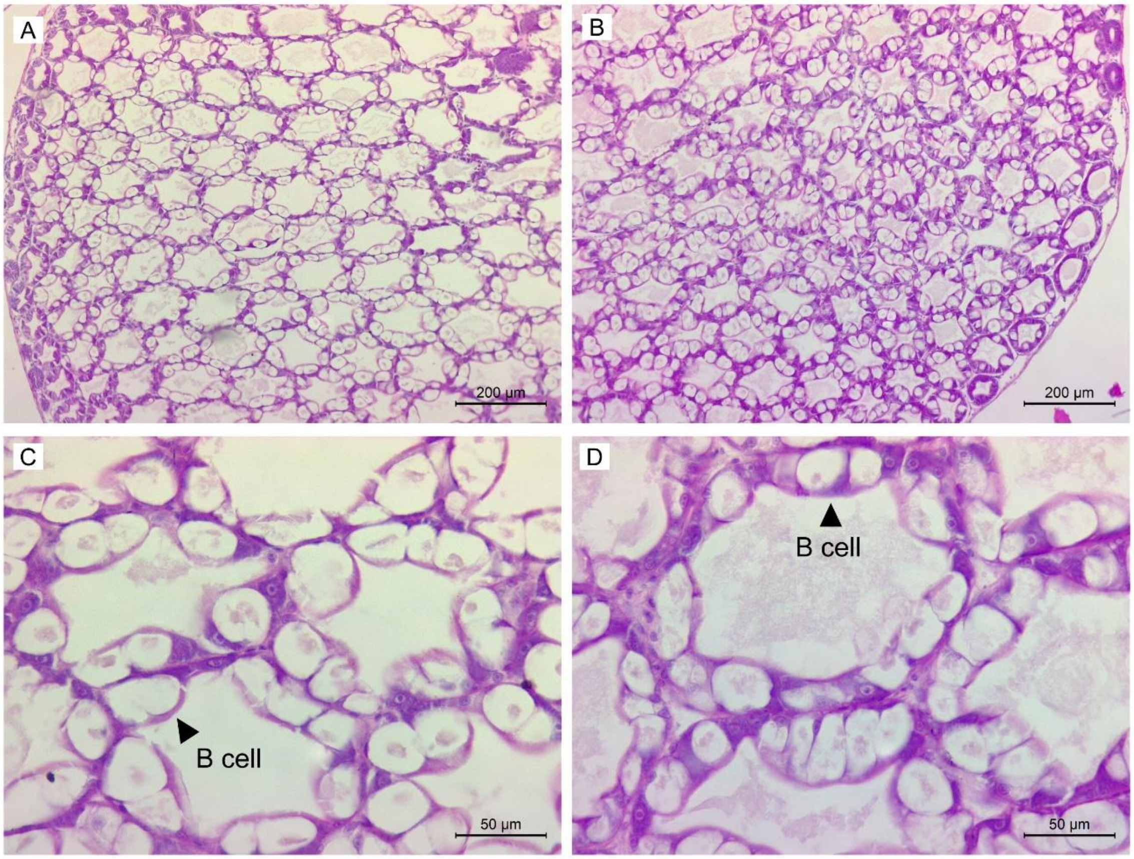

Supplementary Figure S6 Micrographs showing histology of the hepatopancreas (HP) from shrimp in control (A, C) and COHAB (B, D) tanks at 7 days post-cultivation. Tissue sections were stained with hematoxylin and eosin (H&E). Hepatopancreatic tissues from both tanks show similar characteristics. B-cells were observed in both groups.

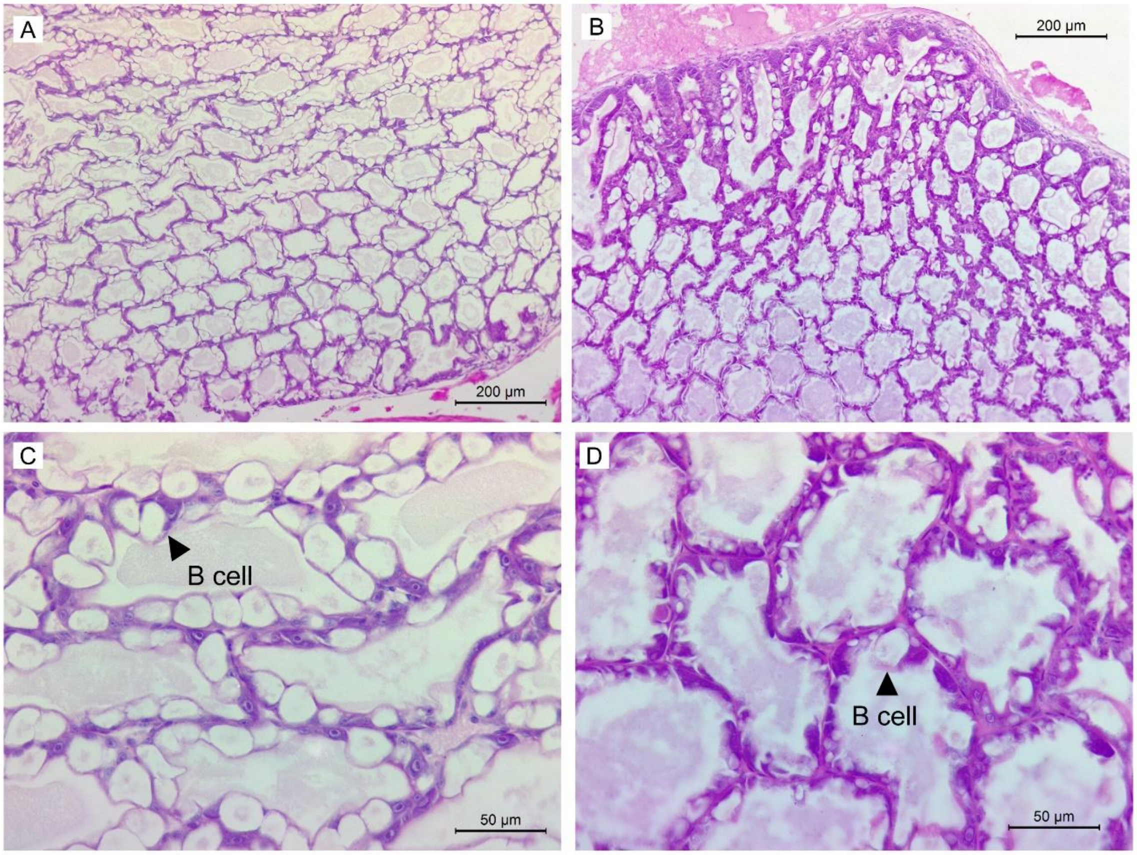

Supplementary Figure S7 Micrographs showing histology of the hepatopancreas (HP) from shrimp in control (A, C) and COHAB (B, D) tanks at 14 days post-cultivation. Tissue sections were stained with hematoxylin and eosin (H&E). Shrimp from the COHAB group showed fewer B-cells compared to the control group, and epithelial atrophy with flattened epithelial cells was observed.

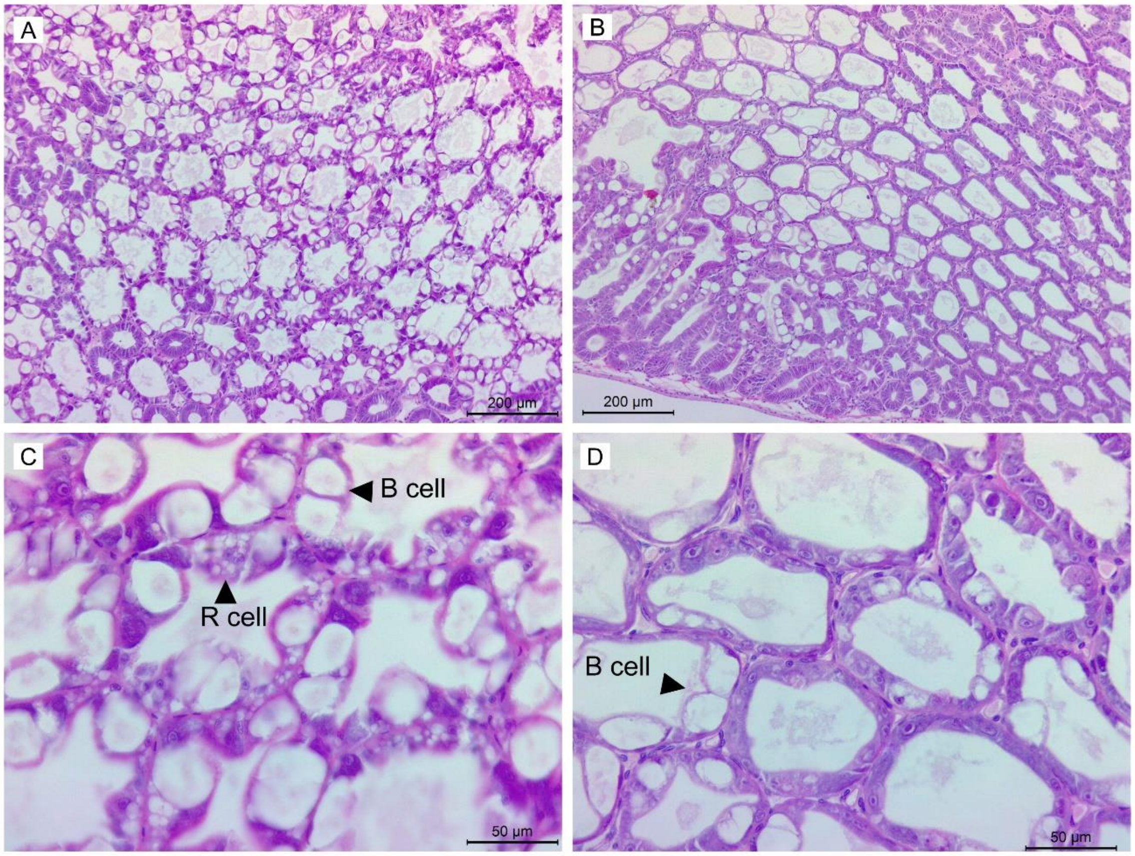

Supplementary Figure S8 Micrographs showing histology of the hepatopancreas (HP) from shrimp in control (A, C) and COHAB (B, D) tanks at 21 days post-cultivation. Tissue sections were stained with hematoxylin and eosin (H&E). R-cells were observed only in the control group, while B-cells were less abundant in the COHAB group. Epithelial atrophy was observed in shrimp from the COHAB group.

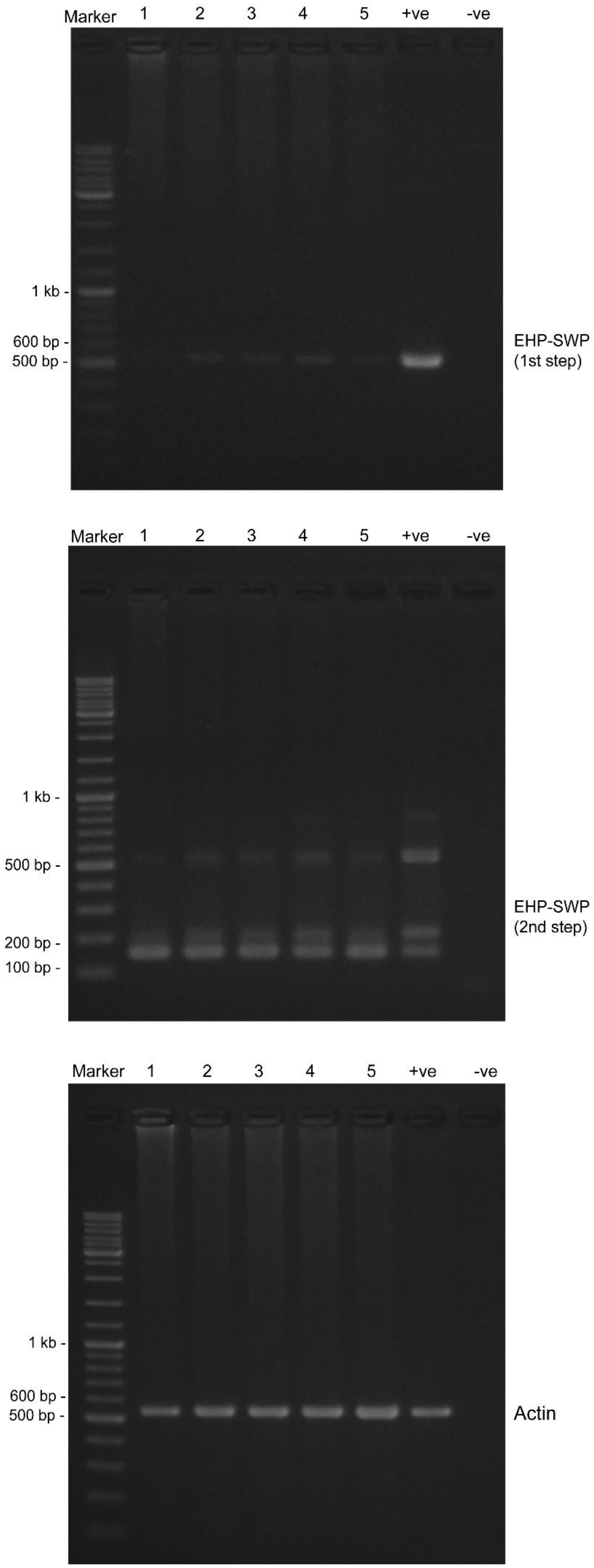

Supplementary Figure S9 Nested PCR detecting the SWP gene in naturally EHP-infected shrimp prior the cohabitation. Five shrimp were randomly selected to test for EHP infection before starting the experiment. Amplicon size of 1st step (top panel), 2nd step (middle panel), and actin (bottom panel) amplification are 514, 148, and 550 bp, respectively. +ve; positive control. –ve; negative control.
